# Arithmetic Training Does Not Improve Approximate Number System Acuity

**DOI:** 10.3389/fpsyg.2016.01634

**Published:** 2016-10-25

**Authors:** Marcus Lindskog, Anders Winman, Leo Poom

**Affiliations:** Department of Psychology, Uppsala UniversityUppsala, Sweden

**Keywords:** approximate number system, arithmetic fluency, training, short-term memory, numbers

## Abstract

The approximate number system (ANS) is thought to support non-symbolic representations of numerical magnitudes in humans. Recently much debate has focused on the causal direction for an observed relation between ANS acuity and arithmetic fluency. Here we investigate if arithmetic training can improve ANS acuity. We show with an experimental training study consisting of six 45-min training sessions that although feedback during arithmetic training improves arithmetic performance substantially, it does not influence ANS acuity. Hence, we find no support for a causal link where symbolic arithmetic training influences ANS acuity. Further, although short-term number memory is likely involved in arithmetic tasks we did not find that short-term memory capacity for numbers, measured by a digit-span test, was effected by arithmetic training. This suggests that the improvement in arithmetic fluency may have occurred independent of short-term memory efficiency, but rather due to long-term memory processes and/or mental calculation strategy development. The theoretical implications of these findings are discussed.

## Introduction

The approximate number system (ANS) is a mechanism believed to mediate our ability to make fast approximate judgments of non-symbolic number in tasks such as deciding without counting which apple tree contain more apples. Large inter-individual differences have been found in ANS acuity (efficiency) and several studies have documented a relation between individual ANS acuity and achievement in symbolic arithmetic (Halberda et al., 2008; Price et al., 2012; Lindskog et al., 2013b; Chen and Li, 2014; Schneider et al., 2016). The causal direction between the ANS and math ability is, however, still under investigation and lively debated (Halberda and Feigenson, 2008; Halberda et al., 2008; Lindskog et al., 2013a; Park and Brannon, 2013; Hyde et al., 2014). Although there is a possibility of a third variable mediating the relation, the dominant views involve a direct causal link. It is, however, not clear if the ANS is a prerequisite for math performance or if math performance influences ANS acuity, or if the relation is bi-directional.

It has been proposed that the ANS lays the foundation for the development of symbolic math (e.g., Dehaene, 1997; Wynn, 1998; Gelman and Gallistel, 2004; Gilmore et al., 2007), which could be the theoretical basis for a relation. Research showing that ANS abilities can be found in several non-human species and in children before familiarization with formal math (Feigenson et al., 2004), and that developmental dyscalculia is associated with ANS impairment (Piazza et al., 2010; Mazzocco et al., 2011), supports this causal direction. Also, in experimental studies training with non-symbolic addition and subtraction has been claimed to enhance math performance (Park and Brannon, 2013, 2014) of adults. For children, however, similar studies have shown mixed results (Obersteiner et al., 2013; Hyde et al., 2014) when investigating the same question. A further finding in line with this position is that pre-verbal number sense in 6-month old infants has been found to predict math ability 3 years later (Starr et al., 2013).

There is also a possibility of a causal link in the reversed direction, that math education or familiarization with symbolic numbers modifies and sharpens the ANS. It is well-known that ANS acuity improves with age (e.g., Halberda et al., 2012). This improvement is generally interpreted in terms of maturational processes. However, it has also been suggested that the improvement may be associated with formal math education, counting, and exposure to arithmetic (Nys et al., 2013). In the majority of studies, this possibility cannot be ruled out because maturational processes and math education are highly confounded in most developed countries. To further investigate this, Nys et al. (2013) studied participants growing up in a Western cultural context, but lacking formal math education. Schooled adults with formal math education in this study had better ANS acuity than unschooled adults. The authors concluded that the acquisition of culturally determined skills, such as math could modify core cognitive competencies in the domain of numeracy. Furthermore, in support of this view Piazza et al. (2013) studied the Mundurucú in the Amazon among who only some had received schooling at adulthood. A significant positive effect of education (specific to numeracy instruction) on ANS acuity was demonstrated after controlling for chronological age. This result suggests that ANS acuity is not fixed by genetic predispositions, but can be altered even in adulthood. It was also observed in this study that in Mundurucú, those not exposed to education stay at the same level as western children of age 6, about the age when they start to receive formal arithmetic education. At higher levels of education the results are mixed. Castronovo and Göbel (2012), for example, found a difference in arithmetic ability between university psychology and mathematics students. However, there was no difference in ANS acuity between these groups, and there was no correlation between mathematical ability and ANS acuity. This study, however, used a paradigm to measure ANS acuity where the stimuli are presented sequentially and separated by a short time interval. Previous research has indicated that such stimulus presentation may not be optimal for measuring relations between ANS acuity and math ability (Lindskog et al., 2013b). Lindskog et al. (2013b) showed that ANS acuity obtained with simultaneous presentations of the two non-symbolic stimuli correlated with arithmetic fluency whereas ANS acuity obtained with the sequential presentation method did not. One possibility is that the sequential method involves memory resources that overshadow the relation between ANS acuity and math performance. Lindskog et al. (2014) therefore used a simultaneous stimulus presentation and examined three university student groups, with varying degree of math content in their university education. These were humanities students with virtually no math content in their education, business students with math related applied content in their education, but without mathematical education *per se*, and math students with explicit formal math education. In their study, Lindskog et al. (2014) found a trend where students taking more mathematics had better ANS acuity. More importantly, a significant improvement in ANS acuity was found in business students as a function of the number of years of higher education in that third year business students outperformed first year students. Because this was a cross-sectional study the difference could possibly be attributed to other group differences or attrition due to student drop out. Nevertheless, the difference remained significant after controlling for general cognitive ability. The results suggest that mere exposure to applied math problems and numbers for business students may have contributed to a refinement of their approximate number system. A mechanism for this refinement could be an increased efficiency with which they process number by an increased distinctiveness in the underlying magnitude representations.

To summarize, previous research has indicated a relation between non-symbolic magnitude discrimination (ANS acuity) and math ability. This relation has been hypothesized to indicate a causal link. The direction of this link has, however, been debated, and it has even been suggested that a bi-directional causal association exits. By and large the research has been correlational in nature, except for a few studies training people on non-symbolic number tasks, and does therefore not allow causal conclusions. To our knowledge, no study has tried to experimentally, with random assignment and control group, investigate the effect of manipulating people’s familiarity with mental manipulation of numbers in terms of basic arithmetic calculation on ANS acuity. Accordingly, the aim of this study was to investigate the possible effect on ANS acuity of sustained exposure to mental arithmetic training with feedback. A transfer effect on arithmetic fluency following an improvement in ANS acuity would have significant theoretical implications for the interpretation of the association between math performance and non-symbolic magnitude processing efficiency.

Approximate number system acuity is, of course, not the only factor related to arithmetic performance. Research shows that short-term memory performance is also such a factor, although the relation is complex and likely depends on several auxiliary factors (Raghubar et al., 2010). Short-term memory, or working memory, also plays an important role in general cognition. Because of this there has been an increased interest in studies with the purpose of investigating effects of training on working memory tasks on broad cognitive changes, purportedly extending to general fluid intelligence, attentional control, reductions in symptoms of ADHD, and decreasing cognitive decline in old age (see e.g., Shipstead et al., 2012; Karbach and Verhaeghen, 2014; Au et al., 2015; but see, Melby-Lervåg and Hulme, 2016). Accordingly, as a secondary aim here we investigated if symbolic arithmetic training (arithmetic fluency) would transfer to short-term memory efficiency for numbers (digit span). Because of the high involvement of short-term memory processes in manipulating and holding numbers in memory during arithmetic calculation it is fully possible that at least part of an improvement in mental arithmetic efficiency by training is due to an accompanied improvement in working memory. If an effect on ANS acuity is found, it is also of interest to determine whether this improvement is dependent on an improved working memory or not.

## Materials and Methods

### Participants

Forty-six participants (10 males, 36 females, *M_age_* = 22.5) were randomly assigned to the training or control condition. All participants were undergraduates from the humanities (e.g., history, anthropology, religion) from Uppsala University with very limited exposure to higher formal math training. They received movie vouchers or course credits for their participation. All participants received an information sheet on the study and provided informed consent before undertaking the study. The regional ethics committee of Uppsala University (Regionala etikprövningsnämnden i Uppsala) approved the study according to the 1964 Declaration of Helsinki.

### Procedure

All participants carried out two tasks during pre- and post-test; a task measuring ANS acuity with an adaptive testing method and a task measuring short-term memory for numbers. Both tasks are described in detail below. Between pre- and post-test participants in the experimental condition carried out six 45-min sessions of targeted arithmetic fluency training. The training sessions were conducted on separate days and are described in detail below. Previous research has shown that it is possible that merely engaging in a non-symbolic numeric discrimination task is sufficient to induce an improvement in arithmetic fluency (Hyde et al., 2014). Accordingly it is possible that merely engaging in an arithmetic fluency task, without any feedback, might be sufficient to induce a change in ANS acuity. We designed our control condition to address this possibility. Participants in the control group therefore, similar to the experimental group, carried out a session of solving arithmetic problems in conjunction with taking part in the pre- and post-test. However, in contrast to the experimental condition, participants in the control group did not receive any feedback. The time between pre- and post-test sessions for the control group was approximately the same as the time interval between these sessions for the experimental group.

#### Adaptive Non-symbolic Number Comparison

An adaptive test based on the ZEST algorithm was used to measure ANS acuity (King-Smith et al., 1994) in pre- and post-test. This method requires fewer trials to achieve an acceptable reliability than the method of constant stimuli often used in this area of research (Lindskog et al., 2013b).

**FIGURE 1:**
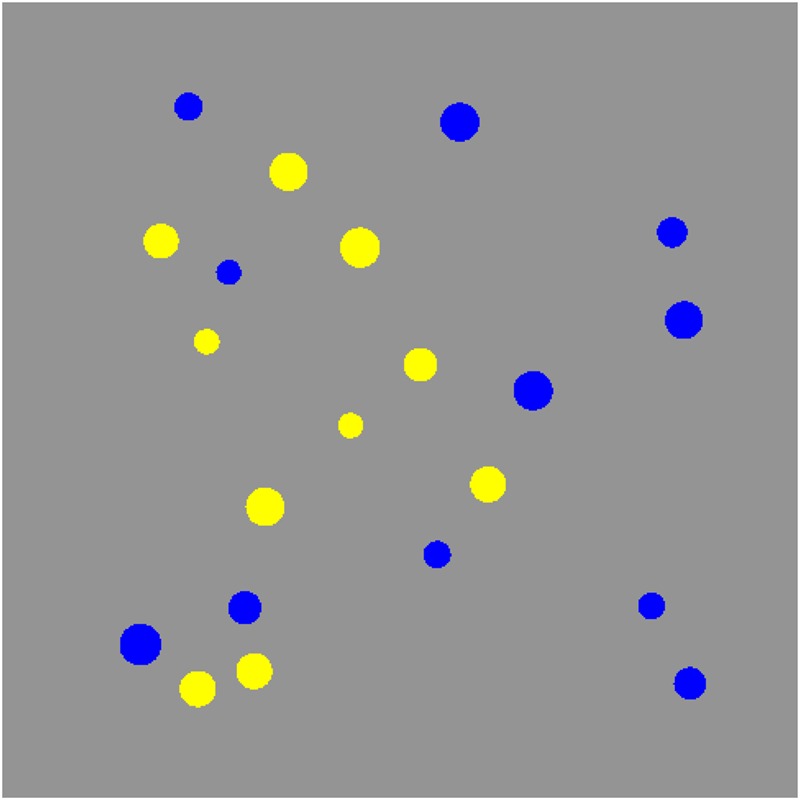
**An example of the numerosity stimuli.** Are there more blue or yellow dots? Participants’ task was to determine there were more blue or yellow dots in the display.

Approximate number system acuity thresholds were measured using non-symbolic representations of numerosity in the form of clusters of spatially intermixed yellow and blue dots presented on an intermediate gray background in each trial (**Figure 1**). Exposure time (300 ms) was short to prevent serial counting of the dots. Dot sizes varied individually in size with a mean radius of 0.35 (range = [0.26, 0.44]) visual degrees at 60 cm viewing distance. Previous research has often used various controls for perceptual variables (e.g., dot size, cumulative area, or convex hull) to minimize the possibility that participants rely on such information rather than numerosity when discriminating between the two sets of dots. Here we used no such controls. The reason for this is a core assumption for the adaptive procedure (outlined below) that the parameters of the psychometric function do not vary from trial to trial. Accordingly, the present algorithm selected difficulty solely on basis of numerosity ratio without controlling for continuous visual cues such as convex hull or cumulative area.

The response was indicated by a press on a yellow or blue marked key on the computer keyboard to indicate what dots were in majority. In both pre- and post-tests participants carried out 240 trials, which has been shown to provide a reliability of 0.80 using this adaptive procedure (Lindskog et al., 2013b). Half of the trials had blue and half had yellow as the more numerous color. No feedback was provided.

Approximate number system threshold for obtaining 80% correct discriminations was estimated by the ZEST algorithm (a modification of the Bayesian QUEST algorithm, King-Smith et al., 1994). The algorithm calculates the stimulus difference for each new stimulus pair (numbers of yellow and blue dots) based on the performance on all earlier trials in the discrimination task. Classical Weber fractions *w*— ΔS/S, where S is a stimulus parameter and ΔS is the inter stimulus difference—was used to quantify the difference between stimulus pairs at each trial. The ZEST algorithm uses all responses in previous trials for optimal Bayesian estimation of the difference between stimuli presented in the next trial to converge on the threshold estimate, *w*, for achieving the desired percentage of correct responses. In short, after each trial the algorithm multiplies a prior probability density function (a prior PDF) of possible *w’s* with a posterior likelihood function of obtaining the response (correct or incorrect) given the prior PDF. The result from this Bayesian integration of likelihood and the prior PDF is an updated density function (a posterior PDF). The mean of the updated PDF is used to determine the stimulus difference of the next trial. The stimulus difference in terms of *w* from the last trial was used as the final estimate. The algorithm simultaneously produced two *w* estimates based on randomly ordered intermixed trials (120 trials each), which were averaged for each participant. The initial prior PDF was a normal distribution of possible Weber fractions with an average of 0.23 and standard deviation 0.58. This initial estimate was derived from previous testing of approximately 200 participants in our lab. A loop was used that searched for the nearest *w* ratio with integer composition of dots with the constraint of an upper total numerosity limit of 33 dots and a minimum number of six dots for the lower numerosity.

#### Short-term Memory

Short-term memory was measured with a digit span test. On each trial, a sequence of digits appeared on a computer screen for 10 s before disappearing. Participants were instructed to memorize and recalled the sequence in the presented order. Starting sequence length was four digits. Sequence length increased with one digit provided that the participant gave the correct answer in three out of four sequences of a particular length. When this criterion was not met, the test was interrupted. The maximum number of digits successfully recalled was used as a measure of the participant’s digit span/working memory capacity.

#### Arithmetic Fluency Training

Participants in the experimental condition received six 45-min sessions of targeted arithmetic fluency training. The first session was conducted directly after the pre-test and the last session was conducted directly prior to the post-test. The other four sessions were conducted on separate days between pre- and post-test. The approximate time between pre- and post-test was 6 days. In each training session participants solved as many arithmetic problems (whole number addition, subtraction, multiplication, and division) as possible. All problems were generated randomly with the constraints described below. The algorithm generating the problems also made sure that an approximately equal number of problems for addition, subtraction, multiplication, and division were generated. Addition and subtraction problems consisted of addends, minuends, and subtrahends of 1–3 digits. For subtraction problems, all differences were positive. Multiplication and division consisted of problems with one single digit factor/divisor and one factor/dividend with 1–3 digits. For division problems, the quotient was always an integer. Directly after answering a problem, participants were shown their own answer together with the correct solution and color-coded feedback (Green = Correct, Red = Wrong).

It is possible that merely engaging in the arithmetic fluency training is sufficient to induce an improvement in arithmetic fluency. We designed the protocol for the control condition to control for this possibility and thereby separate a possible effect of targeted arithmetic fluency training on ANS acuity from an effect introduced by engaging in a task including numerical content. Accordingly, in the control condition participants carried out the same pre- and post-test as in the experimental condition. In addition, participants in the control condition carried out two 45-min session of solving arithmetic problems, one directly after the pre-test and one directly prior to the post-test. These sessions were the same as the corresponding sessions in the experimental condition with the exception that no feedback was given when participants had answered a problem.

## Results

Weber fractions for two participants in the experimental condition (both pre- and post-test) and for two participants in the control condition (post-test only) were lost due to apparatus failure. Data were scanned for outliers (|z| > 3), which resulted in one weber fraction in the post session of the control condition being excluded. The data is avilable at https://osf.io/enrr7.

Each of the two ANS tests was composed of 240 trials. In a study with the aim of assessing psychometric properties, we have previously estimated a reliability approaching 0.80, using this adaptive procedure and amount of trials (Lindskog et al., 2013b). Estimates of the reliability with the present sample suggest a lower reliability of 0.55. Reliability estimates are affected by characteristics such as sample variability. It is realistic to assume that the actual reliability lies somewhere in between these estimates.

### Training Effects on Arithmetic Performance

**FIGURE 2:**
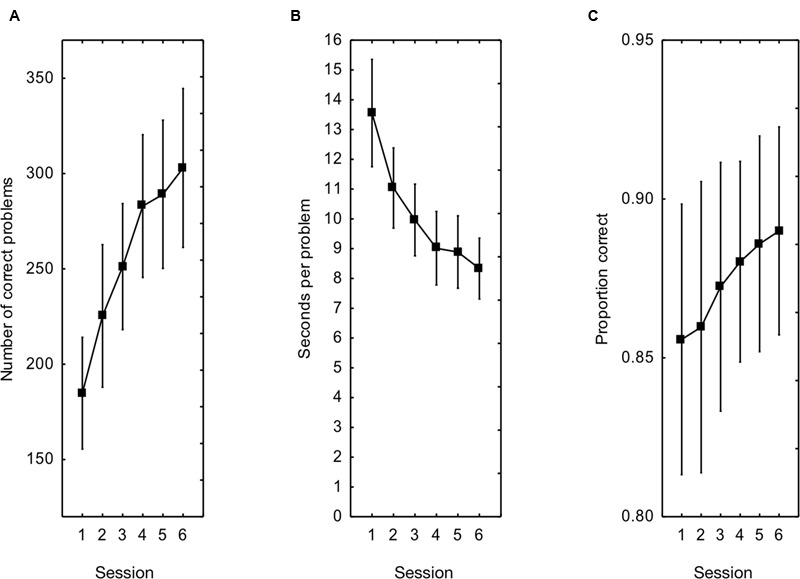
**(A)** The total number of correctly solved tasks during the arithmetic training from sessions one to six. **(B)** The time for solving the tasks. **(C)** The proportion correctly solved tasks. Error bars show 95% CI.

Comparing performance on the first and last session of arithmetic fluency training demonstrated a large improvement by training from an average of 185 to 303 correctly solved tasks, a difference of 118 tasks (**Figure 2A**). A one way repeated measure ANOVA on the total number of correctly solved tasks per session found a significant effect of training, *F*(5, 110) = 57.1, *p* < 0.001, $\eta_{p}^{2}$ = 0.72. A two-way repeated measures ANOVA with training session and operation (addition, subtraction, multiplication, division) as independent variables and percent increase over baseline (Session 1) as dependent variable indicated that learning occurred to a similar degree for all operations [main effect of operation *F*(3, 66) = 1.4, *p* = 0.25] The training session by operation interaction was not significant, *F*(12, 264) < 1. The average percentage increase in problems solved from the first to the last training session was 73%.

The time spent on each problem decreased from 13.6 to 8.3 s/problem, a 40% increase in speed (**Figure 2B**). A one way repeated measure ANOVA on all training sessions found a significant improvement in speed, *F*(5, 110) = 48.79, *p* < 0.001, $\eta_{p}^{2}$ = 0.69. The proportion of attempted problems actually solved correctly increased from 0.86 in the first session to 0.89 in the last session (**Figure 2C**). A one way repeated measure ANOVA on all training sessions found a significant improvement in the proportion correctly solved tasks, *F*(5, 110) = 5.96, *p* < 0.001, $\eta_{p}^{2}$ = 0.21. Thus, there were large effects in the experimental group in terms of number of problems solved correctly, on time spent at each problem and on the accuracy with which problems were solved correctly.

To verify that these performance increases were due to training effects three two-way ANOVAs were calculated with session (first/last) and experimental condition (experimental group/control group) as independent variables and each of the above performance indices as dependent variables. For the number of problems solved correctly there was no main effect of experimental condition, *F*(1, 44) = 1.15, *p* = 0.29, $\eta_{p}^{2}$ = 0.03, but a significant condition by session interaction, *F*(1, 44) = 49.8, *p* < 0.001, $\eta_{p}^{2}$ = 0.53. The latter effect was due to the larger increase in the experimental condition as compared to the control condition (the control group showed a 9% increase due to a mere practice effect). The main effect of session was also statistically significant, *F*(1, 44) = 94.3, *p* < 0.001, $\eta_{p}^{2}$ = 0.68. Tukey’s *post hoc* tests showed that the experimental groups were statistically different at the last session but not at the first, and that the increase in the control condition was not statistically significant (*p* = 0.25).

For the time spent at each problem, there was no main effect of experimental condition, *F*(1, 44) = 1.09, *p* = 0.30, $\eta_{p}^{2}$ = 0.02, but a significant condition by session interaction, *F*(1, 44) = 13.6, *p* < 0.001, $\eta_{p}^{2}$ = 0.24, driven by the larger speed increase in the experimental condition as compared to the control condition (the control group showed a 13% increase due to mere practice). The main effect of session was also statistically significant, *F*(1, 44) = 55.12, *p* < 0.001, $\eta_{p}^{2}$ = 0.57. Tukey’s *post hoc* tests showed that the experimental groups were statistically different at the last session but not at the first, and that the speed increase in the control condition was close to statistical significance (*p* = 0.053).

For the proportion of attempted problems solved correctly, there was no main effect of experimental condition, *F*(1, 44) < 1, but a significant condition by session interaction, *F*(1, 44) = 10.2, *p* = 0.003, $\eta_{p}^{2}$ = 0.19, evidenced by an increase only in the experimental condition (the control group showed a marginal decrease of 1 percentage units). The main effect of session was not statistically significant, *F*(1, 44) = 2.7, *p* = 0.11, $\eta_{p}^{2}$ = 0.06. The above analyses show that the performance increase effects in the experimental condition were indeed due to training with feedback.

### Effects on ANS Acuity and Short-term Memory

**FIGURE 3:**
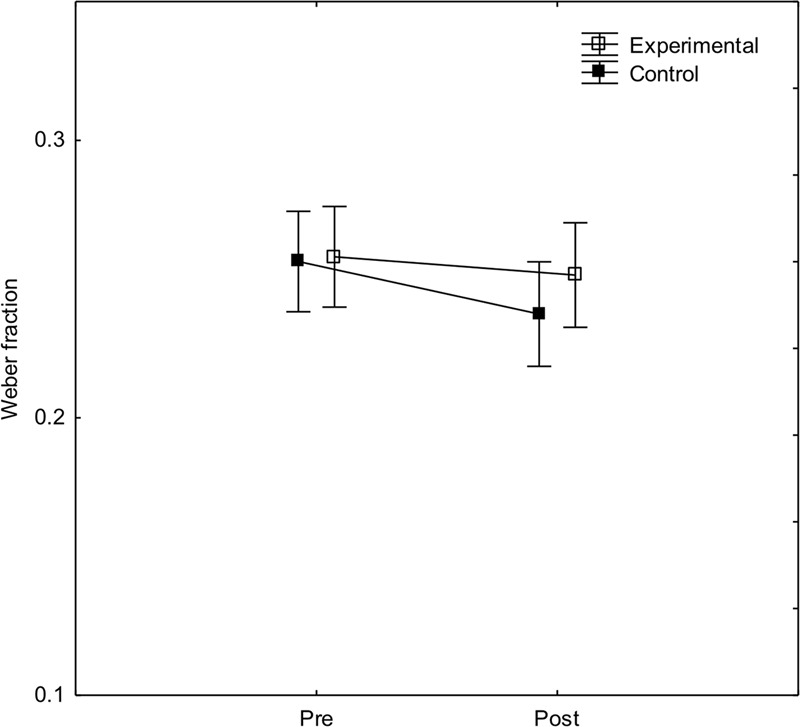
**Weber fractions before and after arithmetic training in the experimental and control conditions, respectively.** Error bars show 95% CI.

To investigate effects of arithmetic fluency training on ANS acuity, a two-way ANOVA with experimental condition and pre-/post-test as independent variables, and weber-fraction as dependent variable was calculated. As can be seen in **Figure 3**, weber fractions were marginally lower (better) in the post-test. There was, however, no main effect of experimental condition, *F*(1, 40) < 1, no interaction between condition and pre-/post-test, *F*(1, 40) < 1, and no main effect of pre-/post-test, *F*(1, 40) = 2.8, *p* = 0.10, $\eta_{p}^{2}$ = 0.07.

**FIGURE 4:**
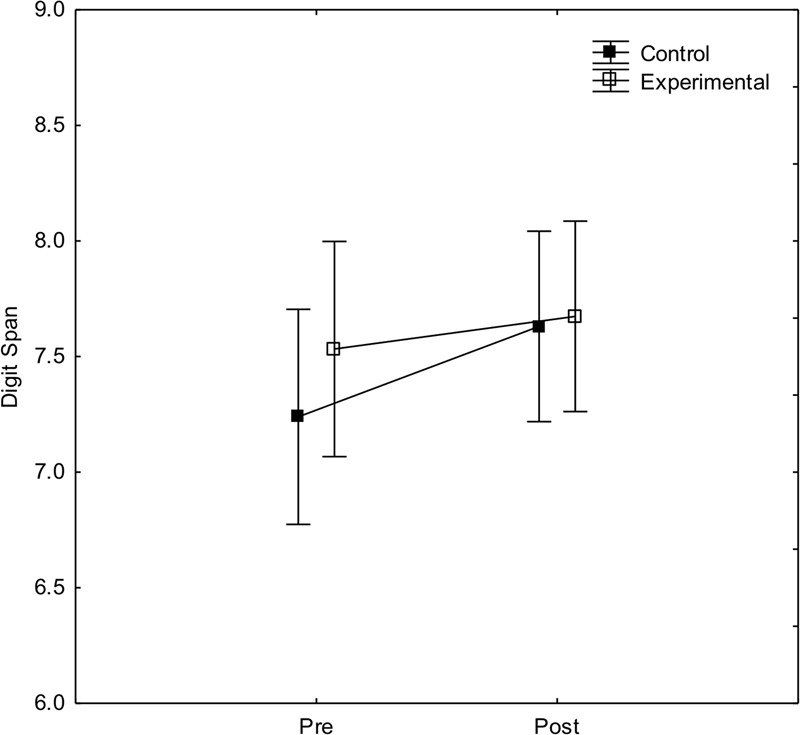
**Performance on the digit span test before and after arithmetic training in the experimental and control condition, respectively.** Error bars show 95% CI.

To investigate effects of arithmetic training on short-term memory (Digit span) a two-way ANOVA with experimental condition and pre-/post-test as independent variables, and digit span as dependent variable was calculated. As can be seen in **Figure 4**, digit span slightly increase in the post-test. The main effect of pre-/post-test was close to significance, *F*(1, 44) = 3.3, *p* = 0.08, $\eta_{p}^{2}$ = 0.07, indicating a small practice effect. There was, however, no main effect of experimental condition, *F*(1, 44) < 1, and no significant interaction between condition and pre-/post-test, *F*(1, 44) < 1.

## Discussion

Recent studies have indicated that non-symbolic arithmetic training transfers to an improvement in math performance (Park and Brannon, 2013, 2014, but see, Lindskog and Winman, 2016). Others have found results that suggest the reverse causal direction (e.g., Piazza et al., 2010; Nys et al., 2013). These results have been inconclusive, being based on correlational data or quasi-experimental designs. Because results (Lindskog et al., 2014) have indicated that math students’ ANS acuity is better than business students and with students from the humanities showing poorer performance we hypothesized that targeted arithmetic fluency training might improve ANS acuity.

We thus investigated if ANS acuity would improve with symbolic arithmetic training. Our results indicated that arithmetic training over six 45 min sessions lead to a large improvement in the number of correctly solved tasks and operation speed. Further, the proportion correctly solved tasks also improved showing that the training effect is not just due to a speed increase, but results in a genuine better precision in arithmetic operations. Also, the lack of improvement in the control condition makes it unlikely that the training results in better performance due to an increased task familiarity. This improvement in arithmetic fluency is noteworthy by itself. Because mental arithmetic ability is highly useful in a society increasingly dependent on numeric information, more exposure to direct mental arithmetic and immediate feedback during math education could contribute to a large performance gain that would be worthwhile with respect to people’s everyday undertakings.

In contrast to previous research showing that non-symbolic arithmetic training transfers to enhancement in math performance (e.g., Park and Brannon, 2013), we found no evidence that arithmetic training *per se* influences ANS acuity. This suggests that there is no causal link from arithmetic fluency to ANS acuity, at least not for adults. A caveat to this conclusion is that previous research (Park and Brannon, 2013, 2014; Hyde et al., 2014) has primarily shown transfer from non-symbolic arithmetic training, rather than non-symbolic comparison training^1^, to math performance. It is thus still possible that the type of training used in the present study might have an effect on non-symbolic arithmetic. Future research should investigate this possibility. As with any training study it could be the case that more extensive training would have resulted in training effects. Our participants, however, received a similar amount of training as participants in previous studies that have indicated training effects in the opposite causal direction (e.g., DeWind and Brannon, 2012; Park and Brannon, 2013, 2014). It is also possible that other kinds of training, for example higher education of conceptual math, involving understanding rather than mental arithmetic practice could bring about effects. Further, it could be argued that a null finding like the present one may be due to an insufficient power. Although a larger sample size is of course desirable it should be noted that our sample size is slightly larger than previous training studies (e.g., Park et al., 2014) showing training effects and that we with the present design have a power of more than 0.75 to detect a large (cohen’s *d* = 0.8) effect size. It should also be noted that the observed means go in a direction contrary to training effects (with a larger reduction of w in the control group than in the experimental group), which further suggest that our results are not due merely to insufficient power. Future studies should examine these possibilities.

A possible limitation to our study is that we did not use a measure of ANS acuity with control for visual cues. Indeed, recent research using a procedure to create stimuli developed by Gebuis and Reynvoet (2011, 2012) (e.g., Gilmore et al., 2013; Szucs et al., 2013) in which not only the more numerous stimuli are larger, but also occupy a larger convex hull on fully congruent trials and vice versa for incongruent stimuli show extreme effects of these variables on difficulty/performance. In some studies, participants do not even perform above chance for incongruent stimuli. Our choice of stimuli was motivated by constraints of the adaptive algorithm, as mentioned above. Thus, a control for visual variables may have interfered with stimulus difficulty, and it is not clear how we would have defined this difficulty in the adaptive algorithm, had we used a control for visual cues. Admittedly, with this procedure, we cannot, exclude the possibility that participants were influenced by, or entirely relied on visual cues in making their judgments. It should be noted, however, that we have in previous work (Lindskog et al., 2013b) established a high converging validity (*r* = 0.88) between the measure of ANS used here and a measure that relies on a more standard way of controlling for visual cues (e. g., Halberda et al., 2008). This makes it likely that our adaptive measure taps the same construct as measures with tasks controlled for cumulative area^2^. It will be an important topic for future research to address to what extent visual cues influences training paradigms of the type used here.

There was no effect of arithmetic training on short-term memory. This is in line with results showing that training on short-term memory resulting in improvement is often tied to strategy and is highly context dependent. What is learnt, then, when arithmetic fluency is improved? A possibility is that learning is a mere strengthening of long-term memory math facts (e. g., 12 + 9 = 21) that are retrieved from memory more rapidly and with less error. Another possibility is that more general “rules” for efficient mental calculation are learned. An interesting venue for future research is to develop ways to measure the exact nature of how human number manipulation is improved by training, which may also have important implications for educational settings.

## Author Contributions

ML and AW developed the study concept. All authors contributed to the study design. Testing and data collection were performed under the supervision of ML and AW. All authors contributed in the data analysis and interpretation. ML, AW, and LP wrote the paper. All authors approved the final version of the paper for submission.

## Conflict of Interest Statement

The authors declare that the research was conducted in the absence of any commercial or financial relationships that could be construed as a potential conflict of interest.

## Footnotes

1 With a “non-symbolic comparison” task participants decide which of two sets of objects that is the more numerous. With a “non-symbolic arithmetic” task, participants view two sets of objects, and decide whether a third set of objects is more or less numerous than either the sum or difference between these two sets.

2 Note however, that there is an ongoing debate about choice of methods to control for visual cues, and that more elaborate methods that control over several dimensions including convex hull result in measures that are apparently not related to measures obtained with more basic controls (see Clayton et al., 2015; Smets et al., 2015).
